# Supplementary material for: A Hybrid Two-Axis Force Sensor for the Mesoscopic Structural Superlubricity Studies
Source: Sensors (Basel). 2019 Aug 5;19(15):3431. doi: 10.3390/s19153431 (PMC6696239; doi:10.3390/s19153431)
Supplement: Supplementary file 1 [file sensors-19-03431-s001.pdf]

Supplementary materials

S1:

Analysis of deformations in teeter-totter structure

1. Primary deformation formats in teeter-totter structure

Three primary deformation formats would perform in teeter-totter structure when applying lateral forces on the tip (also described in ref. [31] cited in the main text) :

1) Torsional deformation about the supporting beams;
2) Bending deformation of the lower part of the mover plate;
3) Collapsing deformation along the supporting beams.

Illustration of the three deformation formats is shown in Figure S1. The corresponding stiffness of the three deformation formats is defined as K_1, K_2 and K_3, respectively. The total stiffness of the system which is the superposition of all modes of deformations is denoted as K. The expressions are:

Torsional stiffness:

$$K_1 = \frac{2G\beta wt^3}{lB^2} \tag{S1}$$

Bending stiffness:

$$K_2 = \frac{EAT^3}{4B^3} \tag{S2}$$

Collapsing stiffness:

$$K_3 = \frac{2Ewt^3}{l^3} \tag{S3}$$

System stiffness:

$$K = \frac{1}{\frac{1}{K_1} + \frac{1}{K_2} + \frac{1}{K_3}} \tag{S4}$$

where, E and G are the elastic and shear modulus of the material, respectively; β, w, t, l are the geometric parameters of the supporting beams, w, t and l are the width, thickness and length of the beam, respectively; A, T and B are the geometric parameters of one half of the mover plate, which are the width, thickness and length of the plate, respectively.

Calculation results of the stiffness of the three deformation formats are shown in Table S1. The results indicate that the torsional stiffness is 2 orders of magnitude smaller than the other two stiffness. Therefore, major deflections are produced by the torsion deformation. Besides, system stiffness of the teeter-tottor structure is nearly equal to the torsional stiffness based on the calculation result. Therefore, the system stiffness of the teeter-totter structure can be simply expressed as the torsional stiffness of the supporting beams.

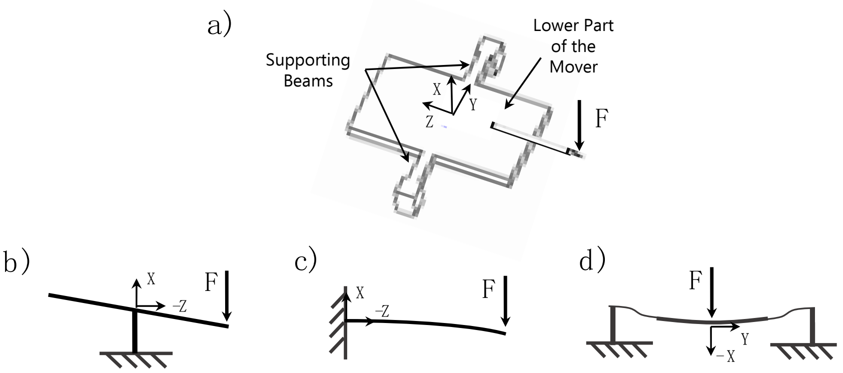

Figure S1. Schematic diagrams of the three deformation formats of the teeter-totter structure. (a) 3D model of the structure; (b) The torsional deformation format from the rotation of the supporting beams; (c) The bending deformation format from the bending of the lower part of the mover; (d) The collapsing deformation format from the bending of the supporting beams.

Table S1. Calculated stiffness of different deformation formats of the teeter-totter structure.

**Structure**	**A (mm)**	**B (mm)**	**T (mm)**	***K_1*[1] (N/m)**	***K_2* (N/m)**	***K_3* (N/m)**	***K* (N/m)**
#1	3	2	0.04	2.27E+01	4.06E+03	2.52E+03	2.23E+01
#2	3	2.5	0.04	2.54E+01	2.08E+03	1.35E+04	2.50E+01
#3	3	2	0.04	3.97E+01	4.06E+03	1.35E+04	3.92E+01
#4	3	2	0.04	4.53E+01	4.06E+03	5.05E+03	4.45E+01
#5	3	2	0.04	6.80E+01	4.06E+03	7.57E+03	6.63E+01

[1] Linear format torsional stiffness.

2. Influence of probe and its junction to the mover

Considering the geometric parameters, stability of the probe is checked by calculating the critical force to produce buckling. Meanwhile, bending stiffness of the probe is calculated. The expression of the critical force F_{cr} and the bending stiffness K_{probe} are:
Critical force to produce buckling:

$$F_{cr} = \frac{\pi^2 EI}{(2L)^2} \tag{S5}$$

Bending stiffness of the probe:

$$K_{probe} = \frac{3EI}{L^3} \tag{S6}$$

where, E is the elastic modulus of the probe material, which is tungsten in this case, with $E =$ 400GPa; $I = \pi R^4/4$ which is decided by the radius of the cross section of the probe R; L is the distance between the point of contact and its junction place.
Calculation results are shown in Table S2. The results present that the probe is in a stable state within the range of use because buckling would happen over several newtons. Besides, the bending stiffness of the tungsten probe is 2 orders of magnitude larger than the torsional stiffness. Thus, it cannot produce a noticeable deformation on the sensor.

UV glue is applied to bound the probe on the mover plate of the teeter-totter part. It is hard to theoretically describe the influence of this junction since the UV glue is a polymer material. However, calibration results present a good repeatability of the sensor with the polymer junction.

Table S2. Calculation results of the critical load and bending stiffness of the probe.

**Structure**	**R** mm	**L** mm	**Fcr** N	**K** N/m
#1	0.15	7	8.00	1.39E+03
#2	0.15	5.6	12.49	2.72E+03
#3	0.15	6.2	10.19	2.00E+03
#4	0.15	7	8.00	1.39E+03
#5	0.15	4.9	16.32	4.05E+03

S2:

Analysis of coupling

1. Coupling issue in the normal force direction

Coupling effect on the normal force may come from two primary reasons: (1) additional deflections related to torsional moments (due to friction); (2) superposition of bending deflection from the teeter-totter structure.

As is depicted in Figure 2 in the main text, normal force sensing structure (the double-cantilever) are designed be sensitive in Z axis. However, lateral forces acting in the X axis can produce moments on the double-cantilever too. The torsional moment would produce additional deflections in the Z axis. We analyzed this issue by calculating the two equivalent stiffness of the normal flexure in both X and Z directions. The results are shown in Figure S2. The equivalent stiffness in the X axis is more than 2 orders of magnitude larger than that in the normal sensitive direction. It indicates that the influence of the torsional moments due to lateral forces on the normal forces can be ignored.

The bending stiffness of the teeter-totter beam is discussed in previous Section 1.1 and the calculated result can be seen in Table S1, $\boldsymbol{K_2}$. Bending stiffness of the teeter-totter beam is 1 order of magnitude larger than the normal stiffness of the double-cantilever. Therefore, deflections caused by the teeter-totter beam are small. For better precision, calibration of the normal force should be conducted with assembly of the teeter-totter part.

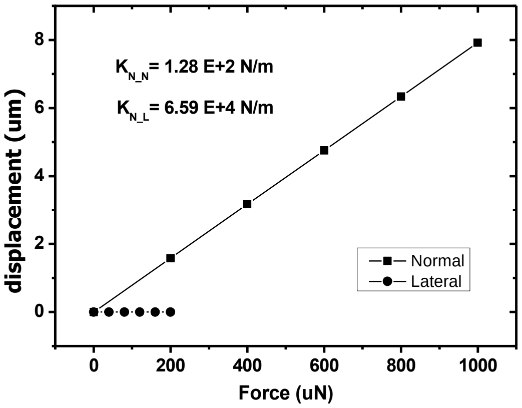

Figure S2. Comparison of the stiffness of double-cantilever in each sensitive and insensitive direction. K_{N_N} is the stiffness related to the normal force, K_{N_L} is the stiffness related to the lateral force.

2. Coupling issue in the lateral force direction

Relationship between the lateral force $\boldsymbol{F}$ and the normal force $\boldsymbol{N}$ can be interpreted as follow:

$$\boldsymbol{F} = \frac{K_t \cdot (-\frac{1}{2}\alpha G + \alpha N + f)}{K_t + \frac{1}{2}GL - NL} \tag{S7}$$

The relationship between $\boldsymbol{N}$ and $\boldsymbol{F}$ shown in the Formula (S7) is not in a linear format. However, we found a highly approximate linear relationship within the load range of several mN. In addition,

we also found that friction force $\boldsymbol{f}$ is related in formula without obvious influence on the ratio $\triangle \boldsymbol{F}/\triangle \boldsymbol{N}$.

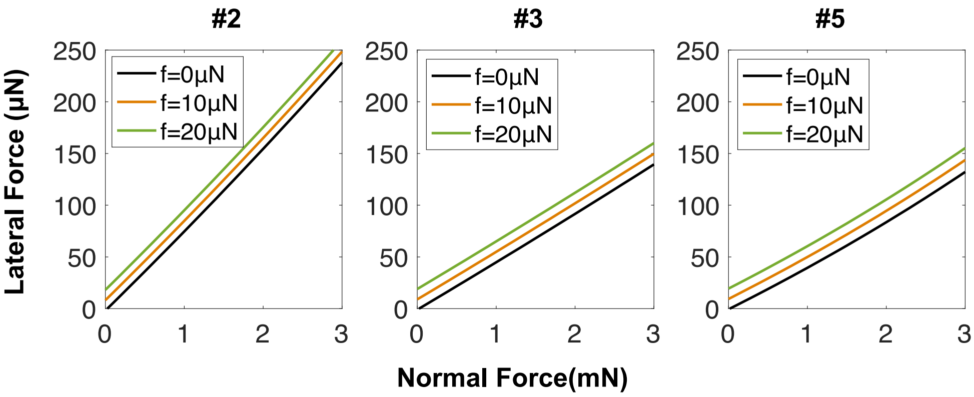

Figure S3. Simulation results of the relationship between the lateral force $\boldsymbol{F}$ and the normal force $\boldsymbol{N}$ under different friction forces $\boldsymbol{f}$.

S3:

Analysis of assembly errors

The assembly errors are analyzed from three projection planes (see the Figure S4).

Figure S4a illustrates the assembly error come from the angle $\boldsymbol{\theta}_{\mathrm{YZ}}$ in the YZ plane. Angle $\boldsymbol{\theta}_{\mathrm{YZ}}$ would cause an additional moment against the X axis on both double-cantilever and teeter-totter structures. Effects related to this error angle are estimated through FEA. Parameters of the weakest teeter-totter beam #1, the normal load 1mN and an exaggerate error $30°$ are used for analysis. The angular displacement produced on the teeter-totter mover by the additional moment is $\sim 2.5 \times 10^{-2}$ degree, which is too small to be concern. The equivalent stiffness of the double-cantilever structure in the normal direction is 1.13×10^{3}, 10 times larger than the normal stiffness K_N described in Equation (1). To suppress the error caused by the angle $\boldsymbol{\theta}_{\mathrm{YZ}}$, a two-points connection configuration with a separated distance is employed in design of the adapter board. Quantitative calibration experiments are shown in Section 4.2 in the main text to further demonstrate assembly errors. The error is controlled within 0.5% in experiments.

Figure S4b demonstrates the assembly error arising from the angle $\boldsymbol{\theta}_{\mathrm{XZ}}$ between the adapter board and the free end of the double-cantilever in the XZ plane. Angle $\boldsymbol{\theta}_{\mathrm{XZ}}$ would cause an additional moment against the Y axis on both double-cantilever and teeter-totter structures. Through FEA, the additional deflections of the double-cantilever structure in the normal direction related to the error is less than 1% of the normal deflections. Besides, for influence on the lateral signals, the error can be subtracted by measuring the friction loop as discussed in Section 3.2 in the main text.

Figure S4c shows the error coming from the angle $\boldsymbol{\theta}_{\mathrm{XY}}$ in the XY plane. In this situation, the measured friction force $\boldsymbol{f}_{\mathrm{X}}$ is a component of the real friction force $\boldsymbol{f}$, expressed as $\boldsymbol{f}_{\mathrm{X}} = \boldsymbol{f} \cdot \boldsymbol{cos\theta}_{\mathrm{XY}}$. Therefore the measured friction force would be smaller than the practical force (e.g. $\boldsymbol{cos30° \approx 0.87,\ cos8° \approx 0.99}$). However, the error angle $\boldsymbol{\theta}_{\mathrm{XY}}$ cannot be large based on the structures of the two assembly parts.

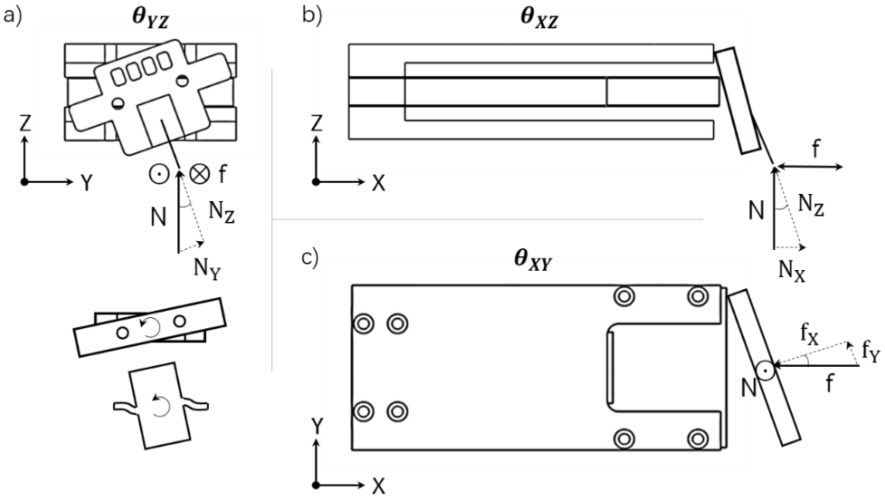

Figure S4. Possible assembly errors in the (a) YZ, (b) XZ and (c) XY plane.

S4:

Detail of the teeter-totter beam

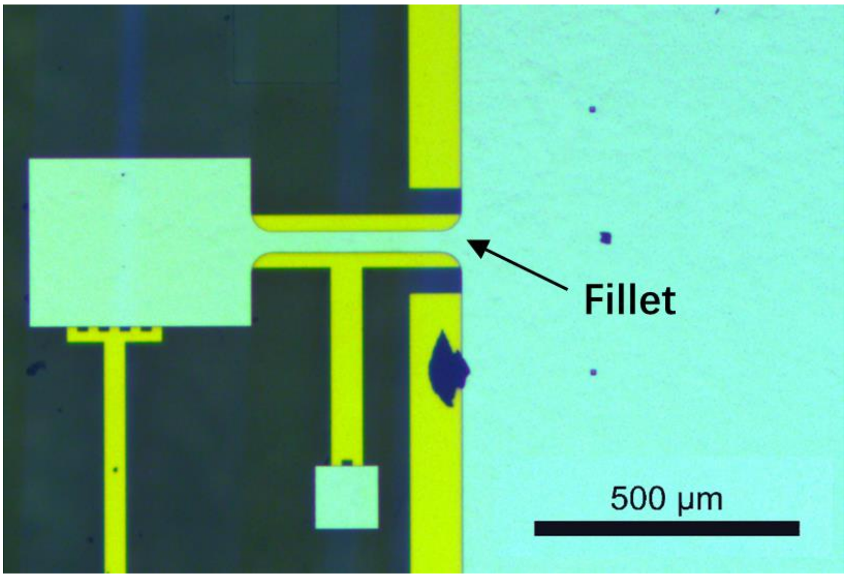

Figure S5. A photo of the teeter-totter beam showing fillets at the beam corners.

S5:

Coupling experiments

Calibration of coupling on the lateral forces

Graphene-graphene interface is applied to calibrate the coupling effect on the lateral forces. The calibration experiment is based on the content discussed in Section 4.2 in the main text. Normal loads are applied in a clear stepping function. Under each load three reciprocating sliding movements in the tangential direction are produced. As is depicted in FigureS6, both normal and lateral force signals show a clear stepping curve. Besides, the friction between the graphene-graphene interface is ultralow.

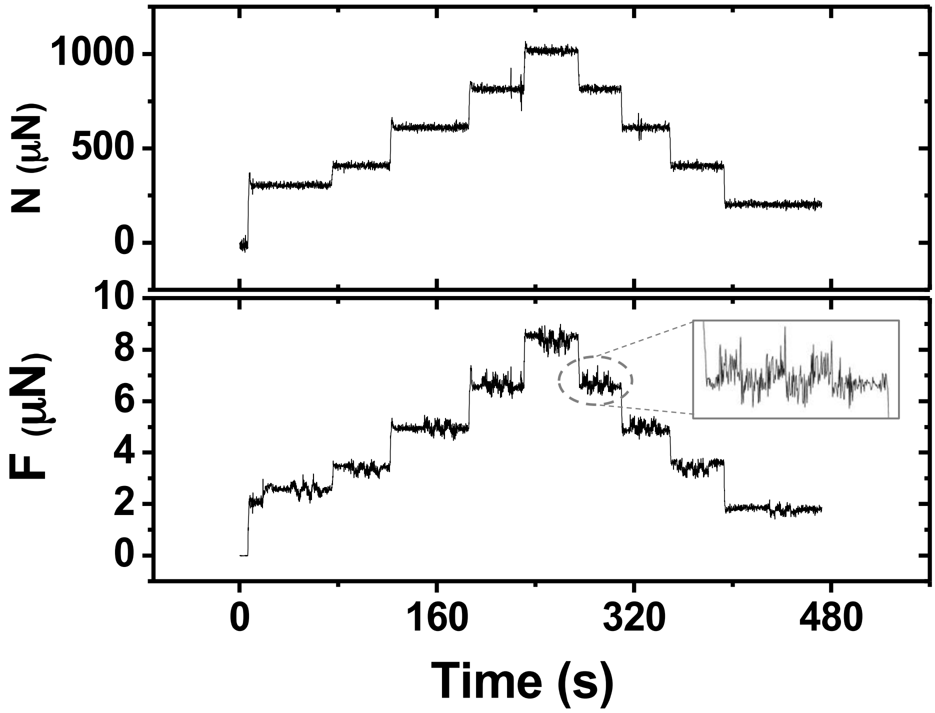

Figure S6. Experiment curves of the coupling calibration. Top figure shows the normal force signals measured by the double-cantilever. Bottom figure shows the lateral force signals measured by the teeter-totter structure. A section of the friction signals during reciprocating motions is magnified in the inset graph.

S6:

More friction experiments

1. Friction experiments with the #2 teeter-totter beam

Frictions between the transferred graphite mesa (5μmx5μm) and the (multilayered) graphene flake are measured by using the #2 teeter-totter beam. The results are shown in Figure S7. Compared to the results done by using the #5 teeter-totter beam, the friction loops measured with the softer flexure are clearer.

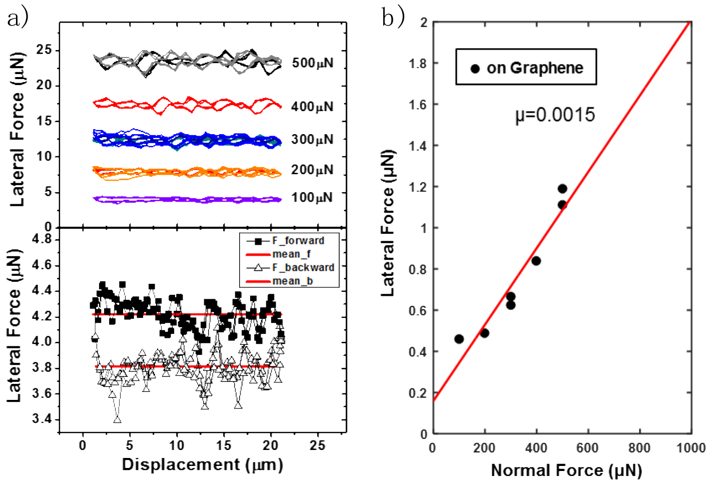

Figure S7. Friction experiments with #2 teeter-totter beam. (a) Friction loops of the graphite-graphene interface; (b) Relationships between the normal forces and the friction forces.

2. Friction experiments with the #5 teeter-totter beam

Friction experiments of directly sliding the sensor probe on different materials are conducted by using the #5 teeter-totter beam. Results are shown in Figure S8. The friction coefficient of silicon (silica) is in the order of 0.01. Whereas, the friction coefficient of graphite is in the order of 0.001.

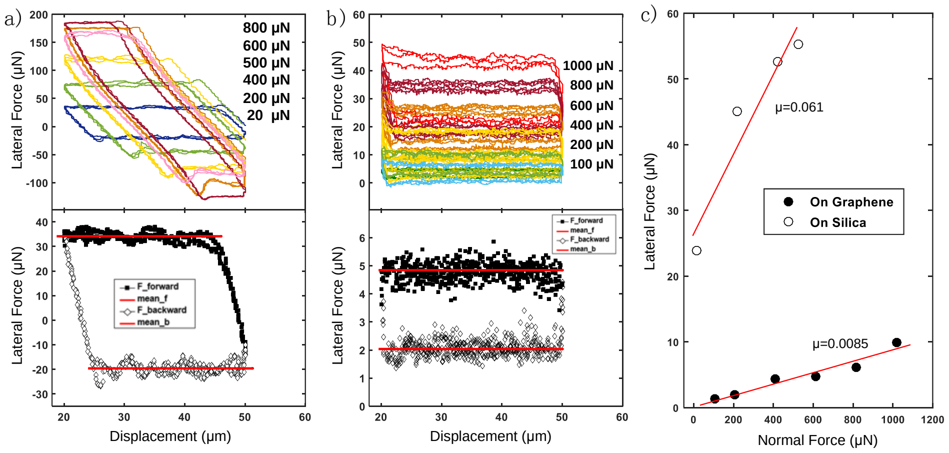

Figure S8. Friction experiments with #5 teeter-totter beam. (a) Friction loops of probe on graphite; (b) Friction loops of probe on silica; (c) Relationships between normal forces and friction forces.
